# Supplementary material for: Inside-out: Antibody-binding reveals potential folding hinge-points within the SARS-CoV-2 replication co-factor nsp9
Source: PLoS One. 2023 Apr 10;18(4):e0283194. doi: 10.1371/journal.pone.0283194 (PMC10085042; doi:10.1371/journal.pone.0283194)
Supplement: S1 Table — (DOCX) [file pone.0283194.s001.docx]

**Supporting Table 1. Data collection and refinement statistics.**

|  | **Nsp9_COV19_:VHH_2Nsp23_** |  |  |  |  |
| --- | --- | --- | --- | --- | --- |
| **Data collection** |  |  |  |  |  |
| PDB code | 8DQU |  |  |  |  |
| Space group | *P6* |  |  |  |  |
| Cell dimensions |  |  |  |  |  |
| *a*, *b*, *c* (Å) | 165.02, 165.02, 46.0 |  |  |  |  |
| (°) | 90, 90, 120 |  |  |  |  |
| Resolution (Å) | 47.6-2.45  (2.58-2.45) |  |  |  |  |
| *R*_pim_^1^ | 4.2 (66.0) |  |  |  |  |
| I/σ_1_ | 10.6 (1.3) |  |  |  |  |
| Completeness (%) | 95.3 (96.1) |  |  |  |  |
| Total N^o.^ observations  N^o.^ unique observations | 93014 (13260)  25484 (3715) |  |  |  |  |
| Multiplicity | 3.6 (3.6) |  |  |  |  |
| CC_1/2_ | 0.998 (0.409) |  |  |  |  |
| **Refinement statistics** |  |  |  |  |  |
| R_factor_ ^2^ (%) | 20.3 |  |  |  |  |
| R_free_ ^3^ (%) | 22.9 |  |  |  |  |
| No. atoms   - Protein - Water - Ligand | 3033  34  5 (SO_4_^2-^) |  |  |  |  |
| Ramachandran plot (%)   - Most favoured - Allowed region - Outlier | 97.5  2.5  0 |  |  |  |  |
| B-factors (Å^2^)   - Protein | 82.2 |  |  |  |  |
| rmsd bonds (Å) | 0.02 |  |  |  |  |
| rmsd angles (°) | 1.50 |  |  |  |  |

^1^ R_p.i.m_ = Σ_hkl_ [1/(N-1)]^1/2^ Σ_i_ | I_hkl, i_ - <I_hkl_> | / Σ_hkl_ <I_hkl_>

^2^ R_factor_ = ( Σ | |F_o_| - |F_c_| | ) / ( Σ |F_o_| ) - for all data except as indicated in footnote 3.

^3^ 5% of data was used for the R_free_ calculation

Values in parentheses refer to the highest resolution bin
