# Supplementary figures and images for: Inside-out: Antibody-binding reveals potential folding hinge-points within the SARS-CoV-2 replication co-factor nsp9
Source: PLoS One. 2023 Apr 10;18(4):e0283194. doi: 10.1371/journal.pone.0283194 (PMC10085042; doi:10.1371/journal.pone.0283194)

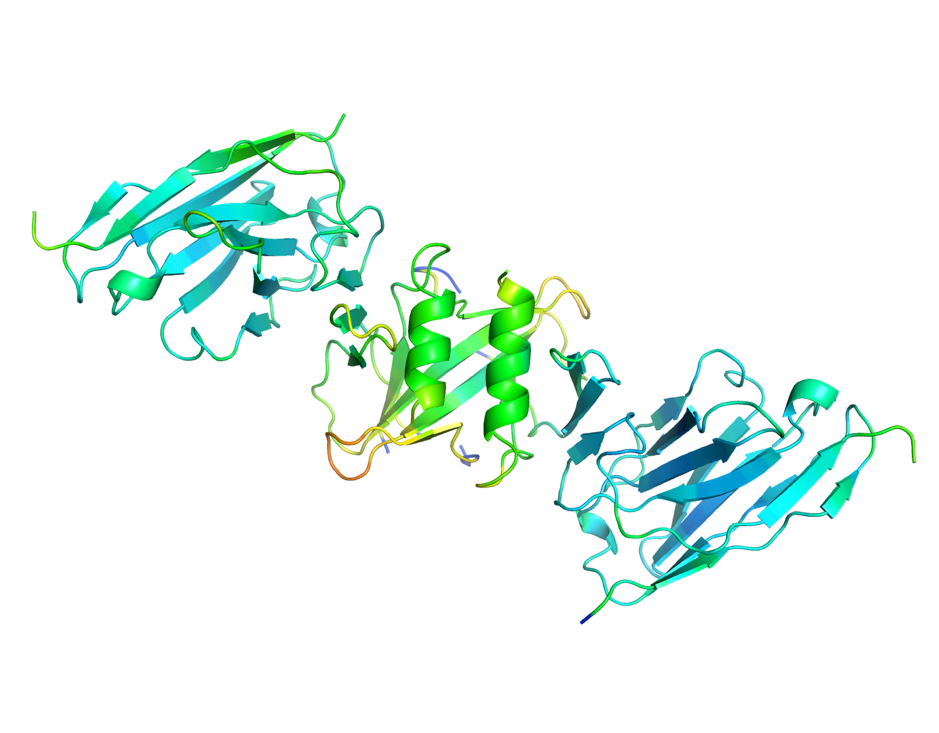

Supplement: S1 Fig — The final model was coloured with a blue-green-red gradient according to the temperature factors of the Cα atoms. (PNG) [file pone.0283194.s002.png]

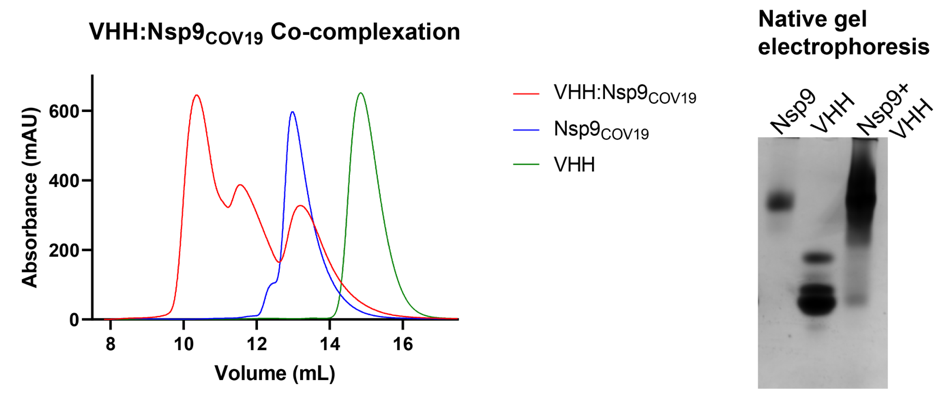

Supplement: S2 Fig — Left: Superdex S75 10/300 gel-filtration chromatograms following injection of ~5mg of VHH2Nsp23, Nsp9COV19 or a pre-incubated mixture. Right: Native gel electrophoresis of ~100μg of VHH2Nsp23, Nsp9COV19 or the complex together. (PNG) [file pone.0283194.s003.png]
