# Supplementary material for: Inside-out: Antibody-binding reveals potential folding hinge-points within the SARS-CoV-2 replication co-factor nsp9
Source: PLoS One. 2023 Apr 10;18(4):e0283194. doi: 10.1371/journal.pone.0283194 (PMC10085042; doi:10.1371/journal.pone.0283194)
Supplement: Attachment — Submitted filename: Nsp9_VHH_Resubmission_v2.docx [file pone.0283194.s004.docx]

**-----------------------------------------------**

**Reviewer #1: This manuscript reports a structure of SARS-CoV-2 nsp9 bound to nanobody VHH2NSP23. Identification of the binding epitope residues will aid future drug development. Changes in the GxxxG helix, which is responsible for Nsp9-NiRAN interactions, moving to the cavity between the s2-s3 and s4-s5 loops, are particularly noteworthy. Understanding the Nsp9/NiRAN interactions is essential to solving the CoV-2 capping mechanism. Although several models have been proposed, the alternative structure of nsp9 revealed in this study may open new possibilities for research. For example, are there native binding partners of nsp9 that can induce these conformational changes? If yes, will it change the capping pathway?**

**The manuscript is clearly written and well illustrated. I suggest a few minor changes**

**1) Page 2 line 4, change ‘Nsp12’ to ‘nsp12’. All viral nsps should be in lowercase throughout the manuscript.**
We thank reviewer1 for the comment, 125 replacements were made – excepting those where Nsp9 is at the beginning of a sentence.

**2) Page 3 line 9, active NMPylation can be supported by Mg2+. Ref “NMPylation and de-NMPylation of SARS-CoV-2 nsp9 by the NiRAN domain, Nucleic Acids Research.**

Agreed we have altered to the following and added reference to end of sentence

“…Mn^2+^-dependent NMPylase…” 🡪 “…Mg^2+^ or Mn^2+^-dependent NMPylase…”

**Line 26, I suggest deleting ‘compromising binding’ here since no data is supporting it in this manuscript.**

Done

**Lines 41-43 are very confusing, please rewrite the sentence. Maybe separate it into two sentences.**

Changed Page 3, line 41 -> “Both components where present in our crystals when run on SDS-PAGE (Fig 1A) but our initial attempts to obtain the antibody-bound nsp9_COV19_ structure was unsuccessful.”

**3) Page 4 lines 13 – 14, does this description correspond to Figure 1C?**
Correct, added a panel callout 🡪“…Gly-93 respectively (Fig 1C)”

**4) Page 7 line 3, if the issue of ‘We note that crystallization conditions can be unusual’ was solved by HSQC, this sentence can be deleted.**

Deleted as recommended.

**line 14, 6 hours. line 15, 50 mM, please check other places. Line 16, in the presence of buffer A? line 35, ‘1:0.5 ratio’? It’s better to specify the ratio of what to what.**
“6hours” -> “6 hours”

14 similar replacements in methods

To ratio added **“**of antibody to nsp9”

**5) ‘VHH2NSP23’ or ‘VHH’? Please choose one and stick to it.**
14 substitutions to standardise this, mainly converting VHH-bound to VHH_2nsp23_-bound

**6) Are ‘nsp9’ and ‘nsp9_COV19_’ different? The use of them is somewhat confusing.**

Added “…in complex with the SARS-CoV-2 nsp9 (we term nsp9_COV19_)

I have used this to distinguish between when we are writing about all coronaviral nsp9 and a specific viral variant. We feel this is important to distinguish as nsp9_SARS_ is very similar to nsp9_COV19_ while nsp9_MERS_ has considerable differences and nsp9_HKU_ is almost unrecognisable. They presumably all control 5’ capping but ideally any NiRAN/nsp9-directed inhibitors should target as wide a range as possible. Viral variant variability also represents potential escape mutants.

**7) Ref 21 has been published.**
Excellent, updated accordingly. We also needed to change page 6, line 44 to Park *et al.* due to the altered first author of the paper.

**8) The names in ‘Author Contributions’ part don’t match the authors in the titles.**
Corrected

**9) Figure 1 legend, buried surface area (BSA); what’s ‘germ’? where is the BSA of CDR1?**

Apologies, changed to the following:

**“**The doughnut chart insert shows the respective contributions to the buried surface area (BSA) for the antibodies CDR-loop and germline residues.”

**And please change the color of CDR1 since green is occupied by nsp9.**

Good idea, altered to orange.

**Use ‘kDa’ for the marker of the SDS-PAGE in (A).**

Added MW (kDa) to Fig 1A

**Not mandatory, but I suggest redoing the SDS-PAGE if possible. The nsp9 band can barely be seen.**

Sorry, this is the SDS-PAGE of the crystal from which the dataset was collected, i.e. it is there to try and show what was in the crystal. I agree it’s an awful gel but was done on a washed 100um^3^ crystal sample.

**In (C), change CDR2 residues to blue.**

Replaced with the following

**10) In figure 2(A), not all of these colored key residues are labeled.**

– Now label Y87

**(B) the ‘s6’ has a different font.**

– fixed in panel B

**(D) what’s the meaning of the color gradient?**

Added to the caption:

“The Ramachandran distances between the two states are mapped onto the canonical nsp9_cov19_ structure using a *white->pink->red* gradient where the upper end of the scale is theoretical maximum”

**(E) what’s the meaning of the underlines?**

Removed underlines – they were highlighting the regions undergoing structural change but are somewhat redundant with panel D.

*Reviewer #2: Pone-D-22-24483
A paper describing the structure of SARS-CoV-2 nsp9 complexed with a nanobody. Nsp9 undergoes a rearrangement with the nanobody binding, disrupting a conserved Beta-sheet and creating new alpha-helices in an inside out pattern. The area changed by the nanobody is thought critical to nsp9 function in the initial viral-mRNA capping reaction, and the authors speculate that this interruption of nsp9 function could be therapeutic in nature. They also speculate that the novel structure could be a naturally occurring as a structural change that occurs in nsp9 during its functions during viral infection. A few comments to address on revision*

**1. The authors speculate that antibodies or nanobodies that have this function of binding nsp9 and inducing the change in structure could be therapeutic during viral infection. They mention in example, therapeutic antibodies that are against spike. I think these anti-nsp9 nanobodies or antibodies would have to delivered intracellularly and make it to the double membrane enclosed replication complex of SARS-CoV-2. I think the authors should point this out and give ideas for such intracellular delivery.**

We agree with this point whole-heartedly! Intracellular delivery of VHHs in live cells is possible but not the preferred option in our opinion. We note the referenced Espositio’s paper had suggested it is a viable possibility though. We see the structural transition induced by the VHH to be informative in itself due to its stabilisation of this new semi-stable secondary form of nsp9. Now we know this nsp9 form can be stabilised we are in the process of trying to induce its formation with cell-permeable cyclic peptides based on the VHHs CDR3 loop. The text has been altered to make this clearer without being too specific about our current work via the following changes:

Page 6, line 4 ->

“Most effort on such antibody-therapeutics have been directed towards virion proteins such as the SARS-CoV-2 Spike, but those binding to other viral proteins have nevertheless shown promise (25).”

Page 6, line 22 ->

“This could be therapeutically advantageous if small molecule reagents could be found that also induce this transition.”

**2. Line 32 on page 5, “guild” should probably be “guide”**

Well spotted, typo has been fixed.

***Reviewer #3: Comments to authors:

In this manuscript, Pan et al. characterized the interactions between SARS-CoV-2 Nsp9 protein with an existing anti-Nsp9 nanobody using X-ray crystallography. The authors solved the crystal structure of the Nsp9-nanobody complex at 2.4 Å resolution. They confirmed that the binding interactions are centered around a Trp-53 within the Nsp9, consistent with the previous NMR-based structural studies of the same complex. They also found that binding the nanobody brings conformational changes in the Nsp9, hampering the Nsp9 binding with its natural substrate NiRAN domain of Nsp12. However, the manuscript has not explicitly discussed the mechanism of such conformational change, which may be a consequence of the nanobody binding.*

The manuscript is well-written and presents an important advancement in the structural understanding of SARS-CoV-2 proteins and their complexes with antibodies, which may help develop alternative strategies for COVID-19 prevention and control. Broadly, conclusions are supported by the experiments, but several points are discussed poorly. For example, the authors said that “the new VHH-bound architecture of Nsp9COV19 represents a novel fold”, but it is unclear how and why it is a “novel” fold of a protein. I expect the authors consider the following points to revise the manuscript before it can be accepted for publication.**

**1) VHH should be defined when used first-time in the text. It is unclear until reading down to the results section.**

Added to Page 3 line 13 ->

“Nanobodies are isolated variable heavy domains from camelid immunoglobulins, also termed VHHs.”

**2) Fig.1A: The labels on top of the bands make them difficult to see. I assumed these were the MW markers.**

Well spotted, this has been fixed.

----------------------------------------------------------------------------------------------
**3) The crystallographic asymmetric unit contained two complexes. It is unclear whether these complexes tend to dimerize in solution. Some tests, such as SEC or native gel electrophoresis, will be helpful.**

Excellent suggestion, we have included the following as Supporting figure 2 which details the shift towards higher MW species.

**Supporting Figure 2 – VHH Co-complexation.**

*Left****:*** Superdex S75 10/300 gel-filtration chromatograms following injection of ~5mg of VHH_2Nsp23,_ Nsp9_COV19_ or a pre-incubated mixture. *Right:* Native gel electrophoresis of ~100μg of VHH_2Nsp23,_ Nsp9_COV19_ or the complex together.

Added: Page 5, line 37 -> “The co-mixture of VHH_2Nsp23_ and Nsp9_COV19_ forms a higher molecular weight species in solution following co-complexation (Supp. Fig. 2).”

**4) What is the affinity of Nsp9 binding with VHH? How does it differ from Nsp9 binding with the NiRAN domain? It may give a clearer perspective on the energetics of Nsp9 deformations caused by the VHH binding.**

This is an excellent question but one I cannot answer at present. The affinity of Nsp9 for the NiRAN domain is hard to measure as this is a multi-component measurement. That is, the native affinity is for the large multi-subunit complex consisting of 5’-RNA:Nsp12:Nsp8 x 2:Nsp7:Nsp13 x 2. Due to the nature of RNA loading within this complex I am not aware of anyone making enough of this 300kDa nucleo-protein structure to consider measuring an affinity. Published EM single-particle reconstructions (Pang *et al* 2022) involve reconstructions of Nsp9-bound forms from tiny amounts of sample. At present the affinity between Nsp9 and RNA/NiRAN domain is probably thought to be low, but the difficultly in ensuring RNA is loaded correctly means I wouldn’t be sure enough to put this in a manuscript.

A transient and conditional association of Nsp9 could make biological sense. 5’-capping must be a tightly regulated interaction *i.e.* a misplaced cap would “waste” an entire viral genome and potentially lead to an immune-visible signature.

**5) Two Nsp9 molecules contact each other heavily within the crystallographic asymmetric unit. Does Nsp9 dimerize in the solution? Does this contribute to the observed deformation of the Nsp9 upon VHH binding? It would be helpful to include this discussion.**

In solution ***in vitro*** Nsp9 adopts a well-characterised homodimeric form first characterised by Sutton *et al.* 2004 for SARS-CoV and which we and others have shown is also adopted for SARS-CoV-2 Nsp9. This homodimeric form has similar monomer structure to the NiRAN-engaged monomeric form of Nsp9 observed in cryo-EM structures.

In this manuscript the VHH-bound homodimeric form of Nsp9 is distinct from the previously published homodimer as shown in the movie. At present it is not currently clear to what extent homodimeric forms of Nsp9 are physiological. Nsp9 requires some coaxing to associate with the NiRAN domain *in vitro* but as described above contributions from the viral RNA enhance binding in single particle cryo-EM analysis. Viral RNA will be more plentiful and stable in an infected cell, distal sections of the viral genome may also enhance Nsp9 binding and we may be missing these at present.

**6) The authors discussed that Nsp9 Trp-53 is the primary binding site for the VHH. However, they did not discuss how Trp53 mutation impacts this binding.**

Tryptophan has a characteristic NMR signature making their cross-peaks easy to identify. Chemical shifts on the Trp-53 peak had previously been used to define it as contributing to the VHH23’s epitope (Esposito *et al. 2021*). This is why we talk about it being central to the epitope and further characterise its interactions. But I the word centrality here to refer to Trp-53 location near the middle of the binding site not to mean it was the sole-contributor to affinity. Other residues mentioned such as Glu-68 and Tyr-66 and Ile-65 make as many interactions upon binding. My assumption would be that binding-affinity is derived from the sum of such interactions. i.e. a simple ala mutation at each site would reduce binding affinity rather than abrogate it. We note that no variations at this site have thus far been observed for SARS-CoV-2 variants and this residue is a Trp in SARS-CoV but not 229E.

To make this point clearer I have changed:

Page 4, line 20 – “centrality” changed to “contribution”

**7) A figure with crystallographic B-factors will be helpful to see rigid and more s**

**Supporting Figure 1 - Overlay of crystallographic B-factor on the Nsp9_COV19_:VHH_2Nsp23_ complex.** The final model was coloured with a blue-green-red gradient according to the temperature factors of the C_α_ atoms.

Page 5, line 34 (Supp. Fig 1.)

**8) Crystallographic data collection and refinement statistics table should be presented (in the manuscript or the SI).**

This is now included in Supp. Table 1 and referenced on page 3, line 37
