# Supplementary material for: Inside-out: Antibody-binding reveals potential folding hinge-points within the SARS-CoV-2 replication co-factor nsp9
Source: PLoS One. 2023 Apr 10;18(4):e0283194. doi: 10.1371/journal.pone.0283194 (PMC10085042; doi:10.1371/journal.pone.0283194)
Supplement: Attachment — Submitted filename: Nsp9_VHH_responseR_v3.docx [file pone.0283194.s005.docx]

Please use the space provided to explain your answers to the questions above. You may also include additional comments for the author, including concerns about dual publication, research ethics, or publication ethics. (Please upload your review as an attachment if it exceeds 20,000 characters)

**Reviewer #3: Authors have addressed most of the issues raised in previous version.**

**However, some of the things authors said "done" in the response letter are not reflected in the revision. For example, the Fig. 1A still contains labels on top of the bands.**

Apologies, version error.

Fig. 1A changed to 🡪
